# Phytate Dephosphorylation Products Also Act as Potent Inhibitors of Calcium Oxalate Crystallization

**DOI:** 10.3390/molecules27175463

**Published:** 2022-08-25

**Authors:** Felix Grases, Antonia Costa-Bauzá, Paula Calvó, Francesca Julià, Jaume Dietrich, Rosa Maria Gomila, Gabriel Martorell, Pilar Sanchis

**Affiliations:** 1 Laboratory of Renal Lithiasis Research, University Institute of Health Sciences Research (IUNICS-IdISBa), University of Balearic Islands, 07122 Palma de Mallorca, Spain; 2 Serveis Cientificotècnics, University of Balearic Islands, 07122 Palma de Mallorca, Spain

**Keywords:** inositol hexaphosphate (InsP_6_; phytate), inositol phosphates (InsPs), crystallization inhibitor, calcium oxalate

## Abstract

Phytate has been classified as an anti-nutrient, but there are no adverse effects from the consumption of a balanced diet with 1 to 2 g of daily phytate (inositol-hexaphosphate, InsP6) as a calcium magnesium salt, the form naturally present in grains. Furthermore, recent research has shown that phytate consumption may prevent pathological calcifications, such as kidney stones and cardiovascular calcifications. However, many endogenous and exogenous enzymes can hydrolyze phytate to lower inositol phosphates (InsPs) that also have biological activity. We performed a controlled hydrolysis of phytate and identified the products (InsPs) using tandem mass spectrometry (MS/MS). The total level of all InsPs was measured using a non-specific methodology. In addition, we evaluated the effects of the InsP6 hydrolysates on calcium oxalate crystallization using scanning electron microscopy and measuring the time needed for the induction of crystallization. Our results indicate that InsP6 and its hydrolysis products functioned as effective inhibitors of calcium oxalate crystallization. Thus, even though InsP6 is hydrolyzed after consumption, the enzymatic products also have the potential to reduce pathological calcifications. Finally, although it is useful to measure the overall level of InsPs in biological fluids, such as urine, there is a need to develop simple analytical methods to quantify the level of individual InsPs.

## 1. Introduction

Researchers first identified inositol-hexaphosphate (InsP_6_, phytate) in 1855–56 and determined its structure in 1914. Mellanby performed the first studies of the possible health effects of phytate during the 1940s by administering sodium phytate to dogs [1]. These early animal experiments indicated that the consumption of a large amount of the sodium salt of phytate with a nutrient-deficient diet caused rickets. This led to the initial classification of phytate as an anti-nutrient. Subsequent studies reported that phytate had the capacity to form complexes with metal cations, and when administered in large doses with an unbalanced diet (deficient in trace elements such as Cu and Zn), it led to malabsorption and nutritional deficiency [2,3,4]. However, more recent studies have shown that the intake of a balanced diet with a moderate amount of phytate (1–2 g/day) in the form of a calcium–magnesium salt led to no adverse effects. In fact, ingesting this amount of phytate is a central part of the "Mediterranean diet" [5]. Studies of the effects of dietary phytate should be performed by supplying it in the form of a calcium magnesium salt and not in the sodium form, as suggested in some recent studies [6].

Recent studies reported that the intake of InsP_6_ can provide numerous interesting and unexpected beneficial effects on health. For example, phytate can function as an antioxidant [7], protect against some types of cancer [8], prevent the development of pathological calcifications [9] and prevent diabetes and its adverse effects [10,11]. Thus, the currently available literature indicates that the intake of phytate has many beneficial effects [12]. InsP_6_ has a strong negative charge, so it is difficult to explain its entry into the body through the gastrointestinal tract. Although similar molecules such as bisphosphonates enter the body by this route (albeit in small amounts), it is thought that there are no specific transporters for phytate, as its absorption is produced by paracellular transport.

The analytical determination of InsP_6_ can be difficult because it does not have any characteristic spectral properties and because of its capacity to form metal complexes, allowing it to bind to multiple metal surfaces and molecules. A further complication is that the body has enzymes that hydrolyze phytate (InsP_6_) to other inositol phosphates (InsP_5_ to InsP_1_) that may also have biological activity. This topic has not been thoroughly studied and has led to various controversies. However, the development of new analytical methodologies that incorporate tandem mass spectrometry (MS/MS) [13,14] has made it possible to reinterpret the results of previous experiments.

Studies of experimental animals and humans have demonstrated that the consumption of InsP_6_ reduced the development of pathological calcifications, such as cardiovascular calcifications, soft tissue calcifications and kidney stones [9]. Since the ingestion of InsP_6_ leads to the formation of less phosphorylated InsPs, it is also necessary to assess the activity of these derivatives as crystallization inhibitors. Polyphosphates with two phosphate groups, such as pyrophosphate and bisphosphonates, are potent inhibitors of the crystallization of calcium salts (phosphates and oxalates) in blood and urine [15,16]. Renal lithiasis is a highly prevalent disease (more than 10% of the population and is increasing), and calcium oxalate lithiasis (in its different types) accounts for around 70–80% of cases. Therefore, the reduction in this type of pathological calcification is important [9].

In the present study, we performed a controlled hydrolysis of phytate and evaluated the hydrolysis process using MS/MS and the total pool of inositol phosphates (InsPs) using a non-specific methodology. We also evaluated for the first time the effects of the different InsP_6_ hydrolysates on inhibiting the crystallization of calcium oxalate (CaOx).

## 2. Materials and Methods

### 2.1. Preparation of InsP_6_ Hydrolysates

A phytate stock solution (1.12 mM) was prepared from phytic acid sodium salt hydrate (68388, Sigma-Aldrich, Schnelldorf, Germany) and was adjusted to a pH of 2 using HCl (0.5 M). Duplicate aliquots (5 mL) were kept in a dry bath at T = 97 °C for 6, 9, 16, 24, 48 or 72 h for hydrolysis.

### 2.2. Identification of InsPs Using MS/MS

Diluted samples (1:1000) were injected into a Q Exactive Orbitrap high-resolution mass spectrometer equipped with a heated electrospray ionization (HESI) probe (Thermo Fisher Scientific, Waltham, USA), which was operated in a negative ionization mode. The temperature of the ion transfer capillary was set to 320 °C, the spray voltage was set to 2.9 kV in negative mode and the S-lens RF level was 50 AU. Direct injection in the full scan acquisition mode over a range of 150 to 700 m/z was performed with a resolution of 140,000.

### 2.3. Crystallization Experiments

The effects of phytate and its hydrolysis products mixture on CaOx crystallization in synthetic urine were assessed using a kinetic turbidimetric system. This system consisted of a spectrometer equipped with a fiber-optic light-guide measuring cell (AvaSpec-ULS2048CL-EVO, Avantes, The Netherlands). Crystallization was assessed at a constant temperature (37 °C) with magnetic stirring (300 rpm).

In these experiments, the synthetic urine solution, obtained by mixing equal volumes of Solution A and Solution B (Table 1), was previously sonicated and adjusted to a pH of 6. Then, 200 mL was transferred to a crystallization flask, and 0.2 mL of phytate stock solution or hydrolyzed mixture was added. When the resulting solution reached a temperature of 37 °C, 2 mL of a sodium oxalate stock solution (5 g/L) was added to induce CaOx crystallization. The time for the induction of CaOx crystallization (which correlates with inhibition of crystallization) was then determined using turbidimetry.

### 2.4. Scanning Electron Microscopy

The morphological and structural characteristics of the CaOx crystals that formed in synthetic urine in the absence and presence of InsP_6_ and its hydrolysis products mixture were examined using scanning electron microscopy (SEM, Hitachi S-3400N, Tokyo, Japan) coupled with RX energy dispersive microanalysis (Bruker AXS XFlash Detector 4010, Berlin, Germany).

### 2.5. Nonspecific Quantification of InsPs

The nonspecific quantification of InsPs, before and after the hydrolysis process, was performed by purification using AG 1-X8 resin and by the formation of InsPs complexes with Al(III), in which InsPs displaced the aluminum-xylenol orange dye, as previously described [17].

### 2.6. Quantification of Inorganic Phosphate

The amount of inorganic phosphate that was liberated during phytate hydrolysis was determined using ammonium molybdate and ascorbic acid (a reducing agent), according to the phosphomolybdate–ascorbic acid method [18]. The absorbance of the blue phosphomolybdous complex was measured at 880 nm.

## 3. Results

**Figure 1:**
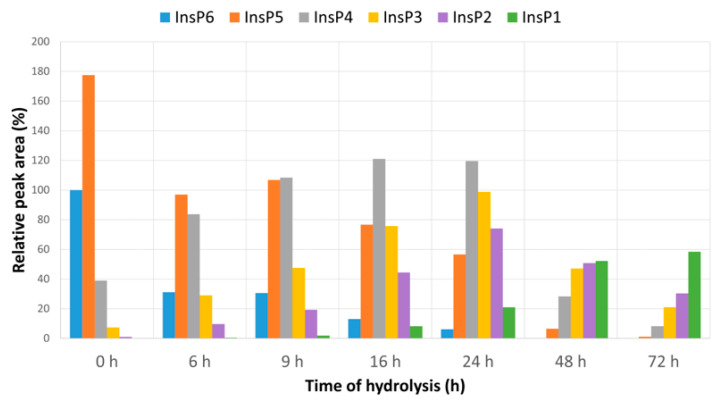
MS signal of the six InsPs after different times of hydrolysis (0 to 72 h) relative to the InsP_6_ signal at 0 h. MS/MS was used for measurements, the absolute level of InsP_6_ at 0 h was 1.12 μM and InsP_6_ was undetectable at 48 and 72 h.

Our MS/MS measurements of a freshly prepared phytate (InsP_6_) solution prior to hydrolysis (0 h in Figure 1) indicated that it was mainly detected as InsP_5_ and that the signal corresponding to InsP_6_ was half that of InsP_5_. This was due to the fact that the MS/MS detector ionization process produced InsP6 fragmentations that mainly involved the loss of a phosphate group. Consequently, the most abundant derivative detected was InsP5, even when the original sample did not contain this compound. Hydrolysis for different times in an acid medium (pH 2) led to changes in the levels of the six different InsPs (Figure 1). Thus, at 6 h, the level of InsP_6_ declined to about one-third of its initial value. InsP_5_ and InsP_4_ accounted for greater percentages of the total, and there were greater levels of InsP_3_ and InsP_2_. At 9 h, the InsP_6_ concentration was slightly lower, and the levels of InsP_4_ and InsP_3_ were slightly greater. At 16 h, the level of InsP_6_ was much lower. The predominant species was InsP_4_, and the level of InsP_1_ increased significantly. At 72 h, the predominant species was InsP_1_, followed by InsP_2_, InsP_3_, InsP_4_ and InsP_5_. InsP_6_ was undetectable at 48 h of hydrolysis.

**Figure 2:**
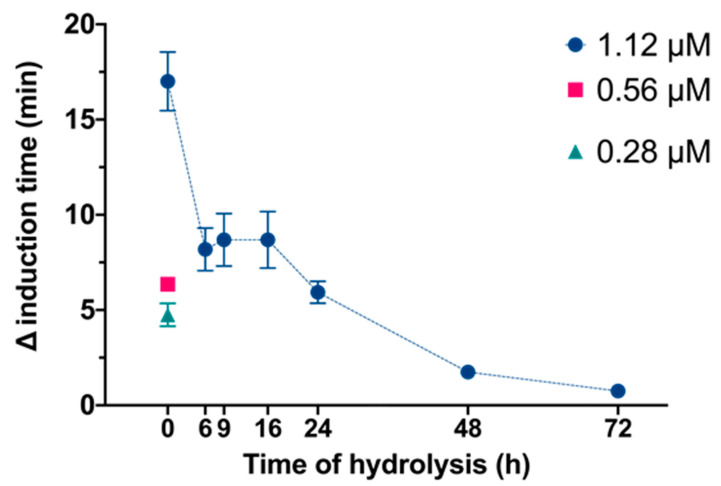
Effect of different InsP_6_ concentrations and InsP_6_ hydrolysates (6 to 72 h) on the increase in the time needed for the induction of CaOx crystallization in synthetic urine. The InsP_6_ levels were 1.12, 0.56 or 0.28 µM for non-hydrolyzed InsP_6_ (0 h), and the total InsPs level was 1.12 µM for hydrolyzed samples. Values are expressed as mean ± SE of three experiments.

We then examined the effects of InsP_6_ and its hydrolysate mixtures on the time needed for the induction of CaOx crystallization in synthetic urine (Figure 2). These results showed that non hydrolyzed phytate at 1.12 μM concentration led to the maximal inhibition of crystallization. Samples with hydrolysis times of 6, 9 and 16 h similarly inhibited CaOx crystallization, producing a higher inhibition than 0.56 μM InsP_6_, even though the presence of InsP_6_ in these samples was much lower, between 30% and 10% of initial concentration (Figure 1). The sample with a hydrolysis time of 24 h had slightly reduced inhibition of crystallization with respect to the 0.56 μM of InsP_6_ and the samples with hydrolysis times of 48 h and 72 h which still inhibited crystallization, but they presented considerably minor effects.

**Figure 3:**
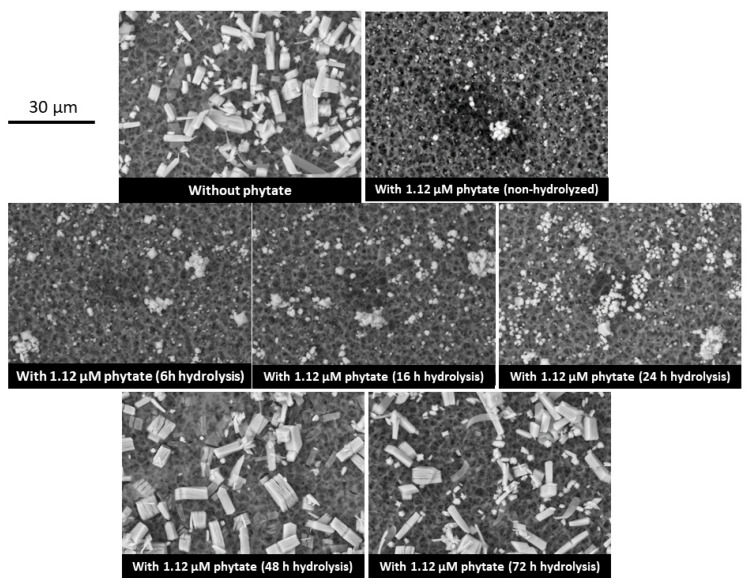
Scanning electron microscopy of CaOx crystals that formed in synthetic urine without phytate, with 1.12 µM phytate (InsP_6_) and with different InsP_6_ hydrolysates (6 to 72 h). Note the presence of COT crystals with a few COM crystals in the experiment without phytate, after 48 h and 72 h of phytate hydrolysis, but not in the presence of non-hydrolyzed phytate or phytate after 6 to 24 h of hydrolysis.

We used SEM to observe the different types of CaOx crystals that formed in synthetic urine in the presence of the different InsP hydrolysates (Figure 3). In the absence of InsP_6_ or its hydrolysates, calcium oxalate trihydrate (COT) crystals were predominant. This product is kinetically favored because the thermodynamically stable form is calcium oxalate monohydrate (COM) [19]. Notably, COT crystals did not form in the presence of InsP_6_ and samples subjected to hydrolysis for 6, 9, 16 or 24 h, indicating significant inhibition of crystallization of all these samples. However, COT crystals formed in the presence of samples that were hydrolyzed for 48 h or 72 h.

We then used a non-specific method to determine the level of total InsPs and free phosphate ions after different times of hydrolysis (Table 2). The non-hydrolyzed sample with phytate at a concentration of 1.12 μM (0 h), when the level of total InsP_6_ was the greatest (Figure 1), led to an InsPs level of 0.82 mM (which corresponds to InsP_6_) and no detectable free phosphate ion. The 6 h hydrolyzed sample had an InsPs level of 0.71 mM and a free phosphate level of 0.45 mM. As the hydrolysis time increased further, the level of InsPs decreased, and the level of the phosphate ionss increased. The 48 and 72 h hydrolyzed samples had detectable InsPs by the non-specific method (0.16 and 0.10 mM respectively), despite the absence of InsP_6_ and a very low amount of InsP_5_ in the hydrolyzed mixtures (Figure 1).

## 4. Discussion

Although the alkaline phosphatases of human mucosal cells apparently do not significantly degrade phytate during intestinal transit, there are other phytases of plant origin that accompany some foods and microbial phytases that can degrade dietary phytate [20]. Furthermore, once absorbed, phytate can also undergo significant dephosphorylation in the liver [21]. Foods rich in phytate that have been processed may contain various InsPs due to the degradation of InsP_6_ [20]. Therefore, the ingestion of InsP_6_ and its subsequent degradation (externally, in the digestive tract, and in the liver) can give rise to a diversity of InsPs in the blood, tissues and urine, as documented in previous studies [22,23]. The variety of these different InsPs could explain why the consumption of InsP_6_ is associated with different health benefits [24]. In particular, these different InsPs may have different specific activities and benefits, as previously suggested to explain the effects of InsPs on rats with type 2 diabetes [25] and on the proliferation of colon carcinoma, in which InsP_4_ and InsP_5_ inhibit the activation of the AKT protein [26]. The less phosphorylated InsPs may be responsible for some of the positive effects of phytate intake on osteoporosis [27], similar to the effects of bisphosphonates. The less phosphorylated InsPs, as bisphosphonates, can also decrease urinary calcium.

A 1972 publication reported that partially hydrolyzed InsP_6_ was a potent inhibitor of the in vitro crystallization of hydroxyapatite [28]. Another 1972 publication reported that partially hydrolyzed InsP_6_ inhibited the in vitro calcification of rat cartilage and that the parenteral injection of partially hydrolyzed InsP_6_ prevented aortic calcification in rats treated with high doses of vitamin D [29]. However, there have been no recent studies of these phenomena or of the effects of different InsPs on CaOx crystallization. Thus, our use of MS/MS provides new information on the effects of these complex mixtures of InsPs. In particular, this study showed that InsP_6_ had the greatest capacity to inhibit CaOx crystallization, but that mixtures of InsP_5_, InsP_4_ and InsP_3_ also functioned as important crystallization inhibitors. Our results confirm that a mixture of InsPs inhibits CaOx crystallization, even when InsP_6_ is not abundant in these mixtures. Therefore, the inhibition of CaOx crystallization in vivo after the consumption of InsP_6_ is likely attributable to InsP_6_ and other hydrolyzed InsPs. Thus, it is very important to consider that the products resulting from InsP6 dephosphorylation are also potent inhibitors of calcium oxalate crystallization.

A limitation of many in vivo studies that examined the beneficial effects of InsP_6_ ingestion is their lack of analytical determination of the different InsPs in biological fluids and tissues. InsP_6_ lacks spectral characteristics that allow easy identification and does not have properties that enable the development of simple analytical methods. An added difficulty is that it is necessary to consider that numerous InsPs products result from InsP_6_ dephosphorylation (the different isomers should also be considered), and these products have similar chemical and biological properties. The most common analytical methods used to quantify InsP_6_ are based on the non-specific evaluation of total inorganic phosphate or complex formation by phosphate groups [17,20], but these procedures obviously require previous purification and separation. Since these methods are nonspecific and the different InsPs have similar properties, the application of these methods may overestimate the amount of InsP_6_ when other InsPs are present [22,23]. In the study of this paper, we assessed the performance of one of these nonspecific methods for measuring InsPs [17] to quantify the content of InsPs of the different hydrolysates, and we then compared the results with those obtained by MS/MS. The results indicated that, as the amount of InsP_6_ decreased because of hydrolysis, the overall amount of InsPs also decreased, and the phosphate ion concentration increased, as can be seen in Table 2. After 24 h of hydrolysis, our MS/MS results indicated that there was very little InsP_6_, and yet the total amount of InsPs was only reduced by about 50% (from 0.82 to 0.39 mM). Only after 72 h of hydrolysis, when the level of total InsPs was very low (with InsP_2_ being the predominant species), the non-specific method indicated a greatly reduced level of total InsPs (0.10 mM).

It is important to note that, in animal experiments in which InsP_6_ was eliminated from the diet, the nonspecific determination of InsPs in the urine indicated extremely low levels [9], and these levels increased significantly when dietary InsP_6_ was re-administered. Other research has shown that experimental animals that were given diets that did not contain InsP6 only excreted small amounts of InsP_2_ in their urine [22]. Therefore, the nonspecific determination of InsPs in urine can provide some useful information. However, there is a need for sensitive and specific methods for the determination of the different InsPs in biological fluids. Although the development of such methods appears difficult, it is clearly important because the different InsPs seem to have different biological effects.

## 5. Conclusions

Our in vitro experiments indicated that the hydrolysis products of InsP_6_ functioned as inhibitors of CaOx crystallization. Thus, even though InsP_6_ is enzymatically hydrolyzed after consumption, its hydrolysis products also inhibit crystallization. This may explain the positive effects of the consumption of phytate on pathological calcifications. Although the determination of total InsPs level can be useful, it is necessary to develop sensitive and specific analytical methods that can quantify each of the hydrolyzed InsPs.

## Figures and Tables

**Table 1:** Composition of synthetic urine. Synthetic urine was obtained by mixing equal volumes of Solution A and Solution B and was sonicated, and the pH was adjusted to 6.0 before starting experiments.

Solution A		Solution B	
Na_2_SO_4_ · 10H_2_O	19.34 mM	NaH_2_PO_4_ · 2H_2_O	15.45 mM
MgSO_4_ · 7H_2_O	5.92 mM	Na_2_HPO_4_ · 12H_2_O	15.64 mM
NH_4_Cl	86.75 mM	NaCl	223.31 mM
KCl	162.69 mM	Na_2_C_2_O_4_	0.6 mM
CaCl_2_	10 mM		

**Table 2:** Level of total InsPs and Pi after different times of hydrolysis. A non-specific method was used to determine InsPs (see Material and Methods).

Time of Hydrolysis (h)	InsPs (mM)	Pi (mM)
0	0.82	0
6	0.71	0.45
9	0.60	0.74
16	0.53	1.3
24	0.39	2.01
48	0.16	4.18
72	0.10	5.41

## Data Availability

Not applicable.
